# University Students’ Psychological Adjustment After Disasters: Investigating the Role of Post-Disaster Stressors, Sense of Community, Social Support Exchanges, and Shifts in Worldviews

**DOI:** 10.3390/bs16030369

**Published:** 2026-03-05

**Authors:** Natalia Jaramillo, Melissa A. Janson, Krzysztof Kaniasty, Annette M. La Greca, Erika D. Felix

**Affiliations:** 1 Division of Adolescent & Young Adult Medicine, Children’s Hospital Los Angeles, Los Angeles, CA 90027, USA; 2 Department of Psychiatry and Behavioral Sciences, University of Washington School of Medicine, Seattle, WA 98195, USA; majanson@uw.edu; 3 Institute of Psychology, Polish Academy of Sciences, 00-378 Warsaw, Poland; kaniasty@iup.edu; 4 Department of Psychology, Indiana University of Pennsylvania, Indiana, PA 15705, USA; 5 Department of Psychology, University of Miami, Coral Gables, FL 33146, USA; 6 Department of Counseling, Clinical, & School Psychology, University of California, Santa Barbara, Santa Barbara, CA 93106, USA

**Keywords:** natural disaster, posttraumatic stress, depression, anxiety, young adults

## Abstract

This multi-university, multi-disaster study examined associations among prior trauma exposure, disaster exposure, and post-disaster life stressors with mental health outcomes, as well as the potential protective roles of a perceived altruistic community, post-disaster social support exchanges, and changes in world beliefs. University students in disaster-affected areas of the mainland United States and Puerto Rico (*N* = 666; 77.5% female; *M* age = 21.26) completed an online survey assessing disaster exposure, post-disaster life stressors, perceptions of community unity, social support exchanges, post-disaster changes in world beliefs, and symptoms of posttraumatic stress (PTSS), depression, and anxiety. Younger age emerged as a risk factor for depression and anxiety, and Black participants reported higher PTSS than White participants. Greater lifetime trauma exposure, experiencing the hurricanes in Puerto Rico or the California wildfires (compared to mainland hurricanes), and reporting more post-disaster life stressors were each associated with elevated PTSS, depression, and anxiety symptoms. In contrast, a stronger sense of an altruistic community was associated with lower levels of these symptoms. More positive post-disaster changes in beliefs about the world were related to lower PTSS and depression, whereas greater involvement in social support exchanges was associated with higher PTSS. Findings underscore the importance of identifying both risk and protective factors that shape young adults’ post-disaster adjustment.

## 1. Introduction

Although much is known about post-disaster adaptation in adults of all ages, less attention has been given to young adults, who may have specific developmental needs (Arnett, 2004). The developmental state of young adulthood (e.g., 18–29 years old) often entails numerous life transitions, including exploring possibilities in relationships, careers, values, and living spaces (Arnett, 2004). Young adults are often pursuing an education to prepare for their desired careers and experiencing a disaster can hinder their ability to excel academically, reduce their job prospects in disaster-affected communities, and increase responsibilities towards family.

Young adults may experience more psychological problems and be less resilient than older adults after a disaster (Bonanno et al., 2010). A scoping review on the psychological outcomes following natural and human-caused disasters identified several mental health consequences experienced among young adults, including posttraumatic stress symptoms (PTSS), general psychological distress, depression, suicidal ideation, anxiety, and sleep and concentration difficulties (O’Donohue et al., 2021). Disaster-exposed young adults with PTSS may also have concerns related to aspects of their identity, relationships, and personal endeavors (e.g., Janson et al., 2024). The current multi-disaster study focused much-needed attention on the experiences of young adults in college or graduate school whose lives were impacted by a disaster. Young adults in higher education represent a developmentally and contextually distinct group whose academic, social integration, and career trajectories may be uniquely disrupted by disasters, yet has been underrepresented in the disaster literature. Centering this population addresses a critical gap in disaster research and advances understanding of how to support their adjustment post-disaster during a key developmental stage. Drawing on the bioecological model (Bronfenbrenner & Morris, 2006), the present study situates disaster exposure within the broader developmental context of emerging adulthood. The framework conceptualizes post-disaster adjustment as embedded within interacting systems, including individual characteristics, contextual stressors, and social-community level aspects. Guided by this theory, the study examined key aspects of disaster exposure influencing post-disaster anxiety, depression, and PTSS, as well as factors such as post-disaster collective helping responses, exchange of social support, and changes in participants’ beliefs about the world.

### 1.1. Risk and Protective Factors Affecting Post-Disaster Adjustment

Racial/Ethnic Factors. Ethno-racially minoritized groups often experience severe psychological outcomes post-disaster compared to those with majority group status (Matsudaira et al., 2021; Norris et al., 2002), suggesting the need to examine this issue among young adults as well. Research conducted with residents from different ethnic backgrounds (e.g., Latinés, non- Latinés, Blacks, and Whites) in South Florida following Hurricane Andrew showed that ethnic groups differed in levels of PTSS (Perilla et al., 2002). Specifically, Latiné participants showed higher posttraumatic stress than Whites and non-Latiné Blacks. Importantly, Perilla et al. (2002) also found that Latinés who preferred speaking Spanish had the highest levels of PTSS (38%) and were more likely to experience intrusion and avoidance symptoms.

Disaster Exposure, Ongoing Life Stressors, and Prior Trauma Exposure. Numerous contextual factors, such as exposure to the disaster, and displacement can influence mental health following disasters (Fussell & Lowe, 2014; Lowe et al., 2013). Factors contributing to post-disaster distress may be influenced by income level, ongoing life stressors, and delays in academic progress as a result of the disaster (Cao et al., 2020). For example, research on low-income young adult parents (ages 18–34) displaced by Hurricane Katrina showed that participants who relocated and those in unstable housing had significantly higher perceived stress (Fussell & Lowe, 2014). Furthermore, (Fussell & Lowe, 2014) examined mental health and general wellness in the aftermath of Hurricane Ike, finding that property loss and collective efficacy predicted wellness for disaster survivors. These findings suggest that factors such as low socioeconomic status, chronic life stressors, and differential exposure to the disaster event influence different pathways to mental health outcomes in the aftermath of disasters (Mao & Agyapong, 2021; O’Donohue et al., 2021).Furthermore, research has showed that prior life stressors and trauma exposures can heighten vulnerability to later stress by amplifying emotional responses (McLaughlin et al., 2010). In the context of disaster research, assessing individuals’ trauma histories is therefore valuable for understanding variation in post-disaster distress beyond the effects of disaster exposure alone.

Post-Disaster Community Factors and Social Support. Community support (i.e., relationship between an individual and a larger community) may play a substantial role in facilitating recovery (McCarty et al., 2022). For example, following Hurricane Ike, a study of middle-aged adults found that levels of community support and subsequent mental health consequences after the hurricane varied across types of communities (West et al., 2013). Specifically, community support served as a buffer against psychological distress among nonurban-residing participants, but not for those residing in an urban area. Community unity, which is feeling a strong sense of belonging and motivation toward mutual support following disasters, may be crucial, given that many young adults live on or commute to college campuses (MacQueen et al., 2001). Hence, strengthening a sense of community in the aftermath of natural disasters may be an effective strategy for promoting their adjustment.

A sense of community during a disaster largely hinges on the exchanges of social support, which often play a central role in protecting against post-disaster psychological distress (Kaniasty, 2020; Kaniasty & Norris, 2009). Social support takes various forms, including emotional reassurance, practical assistance with daily tasks, and the provision of information to navigate the challenges of the disaster (Lakey & Cohen, 2000). In the aftermath of disasters, an altruistic community frequently emerges, characterized by heightened social support exchanges in which individuals both provide and receive emotional and practical assistance (Aldrich, 2012; Bonanno et al., 2010). Understanding this process for young adults, who are possibly away from home for the first time and in a more transitory community than adults who are older and more established in their careers and family lives, is needed to determine if a sense of community is a protective factor.

Disruption of Core Beliefs about the World. Exposure to potentially traumatic stressors such as natural disasters can destabilize core beliefs that ordinarily support a sense of safety, benevolence, and predictability in the world. Disasters pose particularly powerful challenges to the assumptive world (Janoff-Bulman, 1989), calling into question beliefs that people are fundamentally kind and helpful, that positive forces outweigh negative ones, and that life events unfold in an orderly and meaningful manner. In the present study, such disruptions are operationalized as directional changes in world assumptions, defined as the extent to which individuals perceive their beliefs about people, the world, and sources of meaning to have weakened or strengthened following exposure to stressors. Consistent with prior work demonstrating that belief disruption mediates psychological distress (e.g., Matsudaira et al., 2021; Norris & Kaniasty, 1991), these changes are conceptualized as psychologically consequential but not inherently pathological. Both the weakening and strengthening of core assumptions may reflect heightened cognitive engagement with the disaster, rather than clearly adaptive or maladaptive outcomes per se (Cann et al., 2010).

Prior research in disaster contexts is consistent with this distinction. For example, among survivors of the 2004 Indian Ocean tsunami, shifts in religious beliefs, irrespective of direction, were associated with elevated posttraumatic stress symptoms, suggesting that belief change itself may index psychological distress (Hussain et al., 2021). Notably, younger adults were more likely to report a weakening of previously held beliefs, highlighting emerging-adulthood as a developmental period in which worldviews are still consolidating, and therefore particularly susceptible to disruption following disaster exposure. This focus on altered assumptions about benevolence, trustworthiness, and spirituality aligns with broader trauma frameworks emphasizing changes in self-organization and meaning systems under extreme stress (Ford, 1999; World Health Organization, 2022), while remaining conceptually distinct from posttraumatic growth or diminution, which reflect perceived outcomes rather than underlying cognitive reappraisals (Tedeschi & Calhoun, 2004).

### 1.2. Current Study

The adjustment of young adults in the context of disasters warrants closer attention, especially regarding the risk and protective factors that influence their ability to cope effectively. Therefore, it is essential to explore how disaster exposure, subsequent life stressors, and post-disaster factors, including changes in core beliefs, social support exchanges, and a sense of belonging to a post-disaster community, relate to psychological symptoms among diverse samples of young adult disaster survivors.

The current study is part of a multi-university, multi-disaster study that examined the impact of natural disasters on the psychosocial adjustment of young adults. It focused on the California wildfires that occurred between October 2017 and August 2018, hurricanes in Florida, Texas, and Puerto Rico that occurred between August and September 2017, and a wildfire followed by a mudslide in central California in January 2018. The primary goal of this study was to address the following research questions: First, how are prior trauma exposure, disaster exposure, and subsequent life stressors associated with post-disaster levels of anxiety, depression, and posttraumatic stress symptoms among young adults? Second, how are young adults’ sense of post-disaster altruistic community, changes in beliefs about the world and the future, and social support exchanges related to post-disaster mental health outcomes?

## 2. Materials and Methods

Following the hurricane and wildfire seasons of 2017, university students from California, Texas, Florida, and Puerto Rico were recruited for the College Life After Natural Disaster study through research participation pools within their university, departmental emails, and social media posts. Institutional Review Board (IRB) approval was obtained at the lead university, and the remaining universities had an IRB reliance agreement with the lead university IRB. In addition to obtaining informed consent from all participants, procedures were in place to protect participant well-being, including the voluntary nature of participation, the ability to withdraw at any time, and information on accessing their counseling center should participation elicit distress, which was included at the end of the Qualtrics survey. Data were collected using an online Qualtrics survey in English and Spanish. In the mainland U.S., an English survey was distributed three to six months after several hurricanes (Harvey, Irma, Maria, and Jose), wildfires (Atlas, Nuns, Bears, Thomas, Creek, etc.), and a mudslide (Montecito) that occurred in 2017–18. Data collection occurred between March 2018 and December 2018. In Puerto Rico, a Spanish questionnaire was disseminated in November 2018, a little over a year after 2017 when hurricanes Irma, Maria, and Jose occurred, and continued until April 2019. Participants received research credit for their participation or had the choice to opt in for a raffle for the chance to win one of 20 $25.00 Amazon gift cards.

### 2.1. Participants

Participants (*N* = 666) were students at universities affected by a natural disaster throughout the mainland United States (74.6%) and Puerto Rico (25.4%). Approximately 63.8% reported experiencing a hurricane, and 36.2% experienced wildfires. The average time since disaster when the survey was completed was 309.79 days (SD = 148.84), largely due to the time it took to translate the survey into Spanish for participants in Puerto Rico. Most students identified their birth sex as female (77.5%) and their gender identity as woman (76.4%), followed by man (22.4%), then trans- or non-gendered (1.2%). The mean age of the students was 21.26 years old (SD = 4.56). Student race/ethnicity was 13.8% Asian, 4.5% Black or African American, 35.1% White, 35.4% Latiné, and 11.1% Mixed/Other. The majority identified as heterosexual (86.5%). Students were 28.4% freshman, 21.1% sophomores, 16.0% juniors, 24.3% seniors, and 10.2% graduate students.

### 2.2. Measures

All measures were completed as self-reports. Several measures had existing, validated Spanish versions available, such as for the disaster exposure, prior trauma history, and all the mental health constructions. For other constructs (e.g., social support exchanges, core beliefs) translation was undertaken by native Spanish speakers and reviewed by the research colleagues in Puerto Rico to ensure the translation was appropriate to their context. The measures reflected the following constructs.

#### 2.2.1. Disaster Exposure

Participants responded to disaster exposure questions used in prior research (Felix et al., 2011, 2019). Items varied slightly based upon hurricane and wildfire questionnaire versions, but asked whether participants were injured, forced to evacuate, lost their home, an animal, or loved one, experienced financial losses because of the disaster, or whether the disaster damaged/destroyed items of sentimental or emotional value, etc. Response options were 0 = no and 1 = yes. Items on damage to participants’ permanent and college residences were answered on a 5-point scale from 0 (no damage) to 4 (total loss or destruction) and converted to a dichotomous scale (0 = no damage and 1 = any damage) based on prior research (Felix et al., 2019). Hurricane and wildfire exposure experiences were summed separately and eventually combined (as described below) to create a continuous measure of exposure. This initial separation was required because the hurricane, wildfire, and mudslide exposure questions differed in wording and in the total number of items used to assess single and multiple disaster experiences (16–23 items). Wildfire and mudslide experiences were combined, since these events occurred shortly after each other in the affected community. If a participant endorsed an experience for either event, they were coded as yes. If a participant endorsed experiences relating to both the wildfire and the mudslide (e.g., evacuated in both), then they would receive only one yes, representing both disasters. Each disaster-exposure total score (hurricane, only wildfire, or wildfire and mudslide) was transformed into z-scores since the exposure sum could not be compared directly across disaster types due to different item numbers. Z-scores were combined into one disaster exposure variable for use in the analysis.

#### 2.2.2. Life Stressors Since the Disaster

Participants indicated whether they had experienced 11 different life stressors since the disaster. Items were adapted from questions used in prior research following wildfire disasters (Felix et al., 2020) and asked about job changes, moving away, illness or injury to self or a family member, money problems, and relationship problems. Response options were 0 = no, 1 = yes. Total scores were the sum of items, with higher scores representing more post-disaster life stressors.

#### 2.2.3. Prior Trauma History

The Life Events Checklist for DSM-5 (LEC-5; Weathers et al., 2013) assessed exposure to prior trauma. Participants responded 0 = no, 1 = yes to potentially traumatic events that can lead to distress. Within the current study, the list of events was shortened from 16 to 12 items to reduce the overall time to complete the survey. Sample events included experiencing a natural disaster, sexual assault, combat exposure, and more. The LEC-5 has shown strong psychometric properties in terms of good convergence validity with other measures of trauma history, predictive validity with distress, and temporal stability across an average of seven days (Gray et al., 2004). A total score for exposure to prior trauma was created by summing the items.

#### 2.2.4. Sense of Post-Disaster Altruistic Community

Six items assessed the students’ perceptions of how united their local communities were in the first weeks after disaster, their sense of belonging within those communities, and their satisfaction with the help received (see Kaniasty, 2012; e.g., “In the first couple of weeks after [disaster], people united and prior disagreements and differences among people disappeared,” “Even today, I still feel slighted because I believe I received less help and aid than I should have received after the disaster” [reverse coded]). Each item was scored using 4-point scale (0 = strongly disagree, 4 = strongly agree). A total mean score was calculated with higher values indicating a greater sense of post-disaster altruistic community (Cronbach’s α = 0.61).

#### 2.2.5. Social Support Exchanges

Six items, adapted from Kaniasty and Norris (2000), measured the amount of support received from various sources (i.e., family, friends, acquaintances) and support provided to these sources. Three types of support were assessed: tangible help (“helping in the clean-up, giving food, loaning money, offering a place to stay”), informational help (“advice and information, suggesting action to take, helping to understand the situation”), and emotional help (“showing affection and concern, showing understanding of feelings, listening to worries”). Participants were asked how often these exchanges happened within the first two months after disasters (0 = never, 1 = once or twice, 2 = a few times, 3 = many times). A total mean score was created with higher scores representing more engagement in social support exchanges. In the current sample, internal consistency was strong (α = 0.90).

#### 2.2.6. Post-Disaster Change in World Beliefs

Participants reported on the extent to which their views about people and the world became weaker or stronger since the disaster (−2 = became much weaker, 0 = no change, 2 = became much stronger). Guided by a confirmatory factor analyses of the World Assumptions Scale (Janoff-Bulman, 1989) conducted by Elklit et al. (2007), the two highest loading items were selected from the benevolence-of-people subscale (e.g., “people are basically kind and helpful”) and from the benevolence-of-world subscale (e.g., “the good things that happened in this world far outnumber the bad”). These four items were supplemented with two additional items tapping changes in students’ assessments of the importance of their religiosity/spirituality (e.g., “I can rely on my religiosity, and/or my spirituality, to make sense out of my life”). One of the items from the benevolence-of-people subscale had a low correlation with the remaining items; therefore, the change-in-world-beliefs scale was based on the average of five items and showed strong internal consistency in the current study (α = 0.83). A higher (positive) score reflected increased perceptions of safety, benevolence, and predictability in the world, whereas lower (negative) scores indicated beliefs that the world is unsafe, untrustworthy, and unpredictable.

#### 2.2.7. Posttraumatic Stress Symptoms (PTSS)

The 20-item Posttraumatic Stress Disorder (PTSD) Checklist for the DSM-5 (PCL-5) assessed DSM-5 symptoms of PTSD. It is intended to screen for PTSD, monitor symptom change over time, and make a provisional PTSD diagnosis (Weathers et al., 2013). The rating scale is 0–4 for each symptom: not at all, a little bit, moderately, quite a bit, or extremely. Psychometric properties in two studies involving trauma-exposed undergraduate students showed that the PCL-5 has strong internal consistency (α = 0.94), test–retest reliability across testing occasions (r = 0.82), convergent validity (r’s = 0.74 to 0.85), and discriminant validity (r’s = 0.31 to 0.70), demonstrating that the measures’ scores are associated with related constructs (Blevins et al., 2015). Due to a survey construction error, one item was missing from the current sample, and so a mean total score was created based on the 19 available items (range of 0–96). In this sample, Cronbach’s α = 0.92, indicating strong internal consistency.

#### 2.2.8. Depression Symptoms

The nine-item Patient Health Questionnaire-9 (PHQ-9) assessed symptoms of depression (Kroenke et al., 2001). It is based on DSM-IV criteria and has psychometric support for its use in detecting depressive disorders (Kroenke et al., 2001). The PHQ-9 is also considered a valid and reliable measure for use among diverse gender and racial/ethnic U.S. college students (Keum et al., 2018). Participants reported how often they experienced depressive symptoms within the last two weeks (e.g., “feeling down, depressed, or hopeless”) and response options were: 0 (not at all), 1 (several days), 2 (more than half the days), and 3 (nearly every day). Scores were summed to form a total score (range of 0–18) which showed strong internal consistency in the current sample (α = 0.88).

#### 2.2.9. Anxiety

The Generalized Anxiety Disorder Scale (GAD-7) is a self-report screening measure of anxiety (Spitzer et al., 2006). It has good sensitivity and specificity for detecting generalized anxiety, panic, social anxiety, and PTSD (Kroenke et al., 2010). Respondents were asked how often they experienced symptoms of anxiety within the last two weeks (e.g., “feeling nervous, anxious, or on edge”) using a four-point response scale (0 = not at all to 3 = nearly every day). A total score was calculated (range of 0 to 21), with higher scores indicating more anxiety. Internal consistency was strong for the current sample (α = 0.90).

#### 2.2.10. Demographics

A questionnaire assessed demographic characteristics, including age, sex, race/ethnicity, and geographic region. Participants ranged from 18 to 29 years old. Because this was a multi-region study (California wildfires/mudslides, U.S. mainland hurricanes, and Puerto Rico hurricanes), regional information was included as a covariate to account for contextual differences, including time since disaster. Age, sex, and race/ethnicity were also included as covariates in the analyses.

#### 2.2.11. Data Analysis

SPSS 25 software was used for data analysis. Regression models were checked for multicollinearity using Tolerance and Variance Inflation Factors, and no significant overlaps in variance explanation among predictors were found. All study variables met assumptions of normality. Multiple regression analyses were conducted with predictors entered in a theoretically guided order; the results presented reflect the final model including all variables.

## 3. Results

### 3.1. Preliminary Analysis

Correlations and descriptive statistics are displayed in Table 1. Mean levels of PTSS, depression, and anxiety were relatively low, indicating that most participants were not experiencing clinically elevated symptoms at time of assessment. Notable variability, particularly for posttraumatic stress (SD = 10.40) suggests meaningful heterogeneity in responses. Disaster exposure was significantly positively correlated with prior trauma, life stressors since the disaster, social support exchanges, and PTSS, but not with anxiety or depression. Life stressors since the disaster were significantly correlated with all measures of post-disaster mental health and with social-support exchanges. The most common disaster experiences were losing utility services (63.2%), damages to recreational and leisure locations (61.2%), and having to leave or evacuate a residence (47.4%). The most common life stressors since the disaster included having friends move away (32.3%), money problems (30.8%), and difficulty passing classes (28.4%).

### 3.2. Risk and Protective Factors Associated with Mental Health

Table 2 displays the final regression results across the three mental health outcomes. Younger age was associated with higher levels of depression and anxiety symptoms. Identifying as Black, compared to White, predicted greater PTSS. Greater lifetime exposure to traumatic events was consistently associated with higher symptom levels across all three outcomes. Participants who experienced the hurricanes in Puerto Rico or the California wildfires exhibited elevated symptom levels relative to those exposed to mainland hurricanes in Texas or Florida. A higher frequency of life stressors since the disaster further predicted greater symptom severity for all outcomes. A stronger sense of post-disaster altruistic community and more positive shifts in beliefs about the world were associated with lower symptom levels. However, greater involvement in post-disaster social support exchanges was linked to higher PTSS.

## 4. Discussion

There is a significant need to understand the unique challenges and adjustment processes faced by young adults following natural disasters, especially in terms of risk and protective factors related to young adults’ mental health (O’Donohue et al., 2021). This age group is particularly important to study, as they may encounter disasters during a critical developmental stage characterized by the pursuit of higher education, identity formation, life exploration, and goal attainment (Arnett, 2004). As universities contend with disasters affecting their student population, it is critical to understand factors related to post-disaster mental health in this age group. This study examined how prior trauma, disaster experiences, and subsequent life stressors relate to post-disaster levels of anxiety, depression, and PTSS among young adults. Additionally, the potential role of a post-disaster sense of altruistic community, post-disaster social support exchanges, and changes in beliefs about the world in post-disaster functioning were examined.

### 4.1. Age and Race/Ethnicity

Among the university student population, younger participants reported more depression and anxiety symptoms than older participants. For younger adults, this may be when they first left home, whereas older undergraduate and graduate students may have more experience living independently, with more established social connections and greater coping skills than younger students.

In addition, Black participants reported more PTSS compared to White participants, but there was no racial/ethnic difference in levels of anxiety or depression symptoms. Racially minoritized groups may be more vulnerable to psychological consequences following disasters (Norris & Alegria, 2005; Davidson et al., 2013), possibly due to systemic inequities. For example, both Black and Latiné communities are disproportionately more likely to be in areas that fall below environmental quality standards, putting them at increased risk during disasters (Raker et al., 2020). Nonetheless, the relationship between ethnicity and disaster outcomes is often complicated by socioeconomic status, disaster exposure, and other social determinants of health (Bonanno et al., 2010). These findings emphasize the need to support the adjustment of those young adults who may be the most vulnerable following a disaster and point to the need for more research to examine the intersection of natural disasters and mental health inequities.

### 4.2. Prior Trauma Exposure, Disaster Exposure, and Life Stressors Since Disaster

Young adults with a prior trauma history and more life stressors since the disasters reported greater PTSS, depression, and anxiety symptoms. In addition, participants from Puerto Rico had higher PTSS, depression, and anxiety symptoms compared to those who experienced disasters in the mainland U.S. Similarly, participants exposed to both a wildfire and mudslide in California experienced higher symptomatology than those who experienced hurricanes. Exposure to multiple disasters (compared to one) has been linked to greater mental health symptom severity (Leppold et al., 2022) Regions exposed to multiple disasters like Puerto Rico (e.g., Hurricane Maria in 2017, earthquakes in 2019–20) also have had a severe lack of available and accessible mental health services (Rivera et al., 2024).

Notably, disaster exposure, although initially correlated with PTSS, was no longer related once prior trauma and life stressors since the disaster were included in the model. Conceptual models of post-disaster mental health (Hobfoll, 1989; La Greca et al., 2010; Newnham et al., 2022) note that it is not solely disaster exposure, but the increased stressors disasters can engender, that influence mental health in the months and years following disaster. These findings underscore the need for tailored strategies that can address both the immediate and prolonged impacts of disasters, particularly for young adults with pre-existing trauma.

In addition, the differences in symptom severity based on region and disaster support the argument that it is important to account for the type and strength of the disaster, as well as community resources and other contextual and historical factors as they relate to recovery processes. For example, Puerto Rico, an unincorporated territory of the U.S. since 1898, has experienced economic difficulties (resulting from austerity policies implemented in 2016) and sociopolitical tension, in addition to devastating natural disasters. Historically, Puerto Rico has received limited government and humanitarian aid during disaster recovery periods (Garriga-Lopez, 2020; Soto, 2020), which many communities depend on for support post-disaster (Bonanno et al., 2010).

### 4.3. The Role of Sense of an Altruistic Community and Post-Disaster Social Support Exchanges

The study findings also revealed the complex relationship between post-disaster social dynamics and disaster psychological outcomes. Students who experienced a stronger sense of altruistic community in the initial weeks following a disaster consistently exhibited lower levels of psychological symptomatology. These findings align with previous research (see Bonanno et al., 2010; Kaniasty & Urbańska, 2024). For example, Ahumada et al. (2024) found a positive and significant association between bonding social capital (trust in neighbors and individuals a person knows) and subjective well-being among people in Puerto Rico in the aftermath of Hurricane Maria. Additionally, survivors who relied on their immediate social networks reported higher levels of social well-being (Ahumada et al., 2024; Kaniasty & Norris, 2009). Following a disaster, universities might prioritize activities that build a sense of community and mutual support which may help prevent future distress.

Although post-disaster sense of altruistic community was a protective factor, young adult disaster survivors who were more actively involved in giving and receiving social support (mutual post-disaster support exchanges) reported higher posttraumatic stress, but not anxiety or depression. This finding may seem contrary to many studies that have shown that lower levels of social support following a traumatic event are associated with poorer mental health (Lowe & Rhodes, 2013; Zalta et al., 2021). However, the majority of these studies have investigated perceived social support (i.e., reporting a belief that help would be available if needed), which is regarded as the principal facet of social support. Conversely, studies assessing received social support (i.e., reporting actually received help from others in times of need) have produced inconsistent findings. Some studies have documented a clear benefit of greater received support in reducing distress, while many others have found no effects or, in some cases, positive associations between received support and increased mental health problems (see Kaniasty & Norris, 2009; Kaniasty & van der Meulen, 2024).

In the present study, social support exchanges were assessed through questions about both receiving and providing support following the disasters. The positive association between support exchanges and posttraumatic stress symptoms likely indicates that survivors with more symptoms were receiving greater help, reflecting their heightened needs. At the same time, they were also more engaged in helping others, perhaps due to norms of reciprocity and the simple reality that both recipients and providers were affected by the same events. However, this effect was limited, as social support exchanges were not significantly related to symptoms of depression and anxiety. It is also important to recognize that assuming active support roles after collective stressors may impose additional psychological burdens on survivors (Rafaeli & Gleason, 2009; Kaniasty & Norris, 2009).

### 4.4. Change in Beliefs About the World

The study findings revealed that post-disaster strengthening in beliefs of the goodness of the world, people, and the value of spirituality was negatively associated with posttraumatic stress and depressive symptoms, and to a lesser extent with anxiety symptoms (approaching the 0.05 significance level). Meaning making has been identified as a crucial component of psychological adjustment, with prior research supporting a negative, and potentially reciprocal, relationship between shifts in world assumptions and emotional responses to potentially traumatic events (Biram et al., 2024; Janoff-Bulman, 1989; Park, 2016). These results highlight the clinical importance of assessing and addressing changes in beliefs about the world, others, and spirituality as a focus for therapeutic interventions.

### 4.5. Strengths, Limitations, and Future Directions

The current study has many strengths, including conducting research in multiple U.S. regions exposed to various natural hazards, such as wildfires, hurricanes, and the dual challenge of wildfires and mudslides. This broad scope is a departure from the typical focus in disaster research on a single event or geographic region. The recruitment strategy, spanning multiple locations in the U.S. and Puerto Rico, enhanced the generalizability of the findings, as students represented several universities in different regions. The large sample of young adults further adds to the robustness of the study. This approach expands understanding of the diverse psychosocial outcomes that may follow disasters in this population. A range of risk and protective factors were assessed, including nuanced aspects of social support, which universities can leverage as preventive interventions to support students following a disaster.

It is important to acknowledge the study’s limitations. Although lifetime traumatic events were accounted for, this study lacked pre-disaster data on participants’ psychosocial functioning and levels of distress, which may have influenced post-disaster psychological outcomes. As a cross-sectional study, it also cannot capture how risk and protective factors influence mental health over time. The study assessed changes in world beliefs as a mechanism linking disaster exposure to psychological adjustment, but the measure may not have captured all relevant shifts in core beliefs. In addition, only limited data was collected on the sociocultural factors affecting young adults in Puerto Rico impacted by disasters. Further research is needed to gain a deeper understanding of both the sociocultural and disaster contexts and their relationship to the psychosocial adjustment of Puerto Rican young adults.

### 4.6. Implications

Focusing on young adults and their specific developmental needs, during a phase of life marked by identity development, pursuit of education to launch their careers, and exploring romantic relationships, is critical for understanding the association between disaster experiences and psychological well-being. In terms of risk for psychological distress, universities may want to prioritize supportive outreach and connection to services for their younger students and those from minoritized backgrounds, who may be at greater risk in facing disadvantages in disaster exposure, health care access, and recovery services. The students with more prior trauma and additional life stressors in the aftermath of disasters reported more symptomatology. Universities often offer drop-in counseling in the aftermath of crisis events, but additional resources could help address post-disaster stressors, such as emergency housing, easy access to academic advising, academic support, and emergency financial aid.

Importantly, the findings underscore the role of social support (both giving and receiving) and a sense of altruistic community are associated with distress symptoms for young adults. Universities may choose to foster community engagement and promote support systems to support students as they adjust in the aftermath of disasters, but should recognize that the most affected students may be the ones also engaged to a greater extent in exchanging mutual tangible and psychological aid.

The study also highlights the association of post-disaster changes in beliefs about the world and mental health symptomatology. This finding may inform intervention development in targeting young adults’ belief systems and helping them in navigating and adapting to shifts in their worldview following disasters. Universities and campus-wide efforts may benefit from incorporating mental health support services that consider the unique challenges faced by young adults during and after disasters. This could involve awareness programs, counseling services, and community unity-building initiatives. In these ways, universities can support disaster survivors in building resilience and recovering more swiftly in this already challenging phase of life.

## Figures and Tables

**Table 1:** Descriptive statistics and intercorrelations among the study variables (*N* = 666).

Measures	1	2	3	4	5	6	7	8	9
1. Prior Trauma Exposure	----	0.18 **	0.28 **	−0.11 **	0.17 **	−0.09 *	0.28 **	0.23 **	0.22 **
2. Disaster Exposure		----	0.21 **	−0.04	0.21 **	0.03	0.14 **	0.06	0.07
3. Life stressors since disaster			----	−0.07	0.35 **	0.03	0.39 **	0.31 **	0.25 **
4. Sense of altruistic community				----	0.13 **	0.25 **	−0.26 **	−0.14 **	−0.17 **
5. Social support exchanges					----	0.23 **	0.23 **	0.07	0.09 *
6. Post-disaster change in world beliefs						----	−0.12 **	−0.17 **	−0.12 **
7. Posttraumatic Stress							----	0.54 **	0.52 **
8. Depression								----	0.78 **
9. Anxiety									----
Mean	1.84	0.16	2.23	2.22	1.46	0.46	8.68	7.33	6.19
Standard Deviation	1.68	0.98	2.53	0.45	0.85	0.68	10.40	5.80	5.29

* *p* < 0.05. ** *p* < 0.01. Note: statistics for dichotomous variables were omitted; their frequencies are presented in the text.

**Table 2:** Final regression results predicting PTSS, depression, and anxiety (*N* = 666).

Predictors	Posttraumatic Stress β	Depression β	Anxiety β
Age	−0.07	−0.15 ***	−0.09 *
Sex	−0.04	−0.07	0.01
Black vs. White	0.09 *	0.04	0.00
Latiné vs. White	−0.02	−0.05	−0.03
Asian vs. White	0.03	0.01	0.02
Mixed/Other vs. White	−0.03	−0.01	−0.02
Prior trauma exposure	0.13 ***	0.14 ***	0.12 **
Disaster exposure	0.04	−0.00	−0.00
Puerto Rico vs. Mainland hurricanes	0.22 ***	0.28 ***	0.18 **
CA wildfires/mudslides vs. Mainland hurricanes	0.15 ***	0.21 ***	0.23 ***
Life stressors since the disaster	0.25 ***	0.25 ***	0.20 ***
Sense of altruistic community	−0.22 ***	−0.07 *	−0.13 **
Social support exchanges	0.14 ***	0.01	0.06
Post-disaster changes in world beliefs	−0.09 *	−0.12 **	−0.07 †
R^2^	0.291	0.199	0.154
Adjusted R^2^	0.276	0.182	0.136
F	19.07 ***	11.56 ***	8.45 ***

† *p* < 0.06. * *p* < 0.05. ** *p* < 0.01. *** *p* < 0.001.

## Data Availability

Data available upon request.

## Abbreviations

PTSS	Posttraumatic Stress Symptoms
